# Relationship between salusin beta levels and the severity of acute pancreatitis in patients

**DOI:** 10.1097/MD.0000000000038685

**Published:** 2024-06-21

**Authors:** Bulent Albayrak, Emel Ayvaz Guneyin, Muhammet Celik

**Affiliations:** a Department of Gastroenterology, Ataturk University, Erzurum, Turkey; b Department of İnternal Medicine, Ataturk University, Erzurum, Turkey; c Department of Biochemistry, Ataturk University, Erzurum, Turkey.

**Keywords:** salusin beta levels, severe acute pancreatitis

## Abstract

**Background:**

This study aimed to evaluate the significance of serum salusin beta (SAL-β) levels in predicting the severity of acute pancreatitis (AP) in patients diagnosed with this condition and to assess its relationship with disease and prognosis.

**Methods:**

Sixty-four patients between 18 and 100 years of age diagnosed with AP, were included in the study. Patients were categorized into 3 groups based on the Revised Atlanta Classification: mild, moderate, and severe AP. Eighteen healthy adults were included as the control group. Sex, age, height, weight, presence of additional diseases, laboratory results, imaging findings, levels of white blood cells, neutrophil-lymphocyte ratio, mean platelet volume, amylase, lipase, sensitive C-reactive protein, sedimentation, and serum SAL-β were measured and recorded. SAL-β levels were reevaluated on the third day of hospitalization.

**Results:**

The average age of the patients included in the study was 62.66 ± 17.67. Gallstones were present in 64.1% of the patients. The difference in the SAL-β averages on the 1st and 3rd days was statistically significant (*P* < .05). On the first day, the SAL-β averages of those with severe Atlanta scores were higher than those with mild and moderate Atlanta severity. Similarly, on the third day, the SAL-β averages of those with severe Atlanta scores were higher than those with mild and moderate Atlanta severity. According to receiver operating characteristic analysis using the Youden index, the cutoff value for SAL-β for severe pancreatitis was 178.8 pg/mL on the 1st day and 207.5 pg/mL on the 3rd day.

**Conclusion:**

SAL-β can be used to detect and monitor severe pancreatitis. Further extensive clinical studies with larger case series are needed.

## 1. Introduction

Acute pancreatitis (AP) is a clinical condition characterized by damage to exocrine pancreatic acinar cells caused by enzymes and the resulting inflammation. Its severity can vary from self-limiting mild pancreatic edema to systemic inflammation leading to pancreatic necrosis, organ failure, and death. Although many factors are associated with AP, gallstones and excessive alcohol use account for 80% of the cases. Determining the severity of AP is crucial for predicting mortality and identifying the subset of patients who will benefit from aggressive treatment. Imaging methods, scoring systems, and biochemical parameters were used to predict severity. Pancreatitis severity is commonly classified radiologically according to the Revised Atlanta Classification.^[1–5]^

Salusins, salusin alpha, and salusin beta (SAL-β) are soluble peptide hormones processed from the same precursor peptide. Salusins are secreted in blood vessels, monocytes, and macrophages, and are found in human body fluids. They have various functions, including vascular inflammation, modulation of the cardiovascular system, cytokine function, and oxidative damage, and act as endogenous neuropeptides. In addition, they stimulate the proliferation of vascular smooth muscle cells (VSMCs) and fibroblasts. In particular, SAL-β has various functions such as cytokine function, modulation of vascular inflammation, and oxidative damage. Recent studies have shown that SAL-β may promote inflammation by increasing activation of the nuclear factor kappa B signaling pathway.^[6–8]^ This study aimed to evaluate the importance of serum SAL-β levels in predicting the severity of AP and its relationship with disease prognosis in patients diagnosed with AP.

## 2. Materials and methods

This prospective study included 64 patients diagnosed with AP, aged between 18 and 100, who were admitted to Atatürk University from July 2022 to May 2023. Patients were categorized into 3 groups based on the Revised Atlanta Classification: mild, moderate, and severe AP. Eighteen healthy adults were included as the control group. The mean age of the control group was 60.13, body mass index was 26.02, there was no smoking, no alcohol use, and no additional diseases, and laboratory results were normal values. Patient’s sex, age, height, weight, and the presence of additional diseases, laboratory results, imaging findings, white blood cell (WBC) count, neutrophil-lymphocyte ratio (NLR), mean platelet volume (MPV), amylase, lipase, sensitive C-reactive protein (CRP), sedimentation, body mass index, bedside index of severity in AP scores, sepsis and septic shock criteria scores, and serum SAL-β were measured and recorded. These parameters were re-measured and recorded from blood samples obtained on the third day of hospitalization. Patients with chronic pancreatitis, chronic lung disease, symptomatic heart failure, chronic renal failure, and patients who did not agree to participate in the study were not included in the study.

SAL-β levels in the serum samples were measured using a commercial kit (Human Salusin-β ELISA KIT, Catalog No. NE010127201, Lot No. 211008, Nephente Research Technologies, Gebze/Kocaeli, Turkey) and the ELISA method according to the manufacturer’s guidelines. The intra and inter-assay values of the test were <8% and <10, respectively. The results are presented in pg/mL.

Treatment planning was conducted for the etiology of AP in the enrolled patients. After hospitalization, oral intake was discontinued; appropriate hydration therapy, nasal oxygen support, and analgesic treatment were initiated; and vital signs were closely monitored. Only 1 patient diagnosed with severe pancreatitis died. Ethical approval for this study was obtained from the Ethics Committee of the Ataturk University Faculty of Medicine, with decision number 110 of session number 3 and dated 31.03.2022.

### 2.1. Statistical analyses

Data were analyzed using SPSS for Windows 22 (IBM Corp., Armonk). In addition to numbers, percentages, minimum and maximum values, mean and standard deviations, independent samples *t-test*, and analysis of variance were applied for normally distributed measurements. For non-normally distributed measurements, Mann Whitney *U*, Kruskal–Wallis, and Spearman correlation analyses were used. In comparing paired groups, the paired-sample *t-test* was used for normally distributed data, and the Willcoxon test was used for non-normally distributed data. receiver operating characteristic analysis and the Youden Index were used to determine the sensitivity and specificity of SAL-β day 1 and day 3 levels in the presence of severe pancreatitis (Revised Atlanta Scoring) and to predict the cutoff value.

## 3. Results

The mean age of patients included in the study was 62.66 ± 17.67 years. Of the participants, 57.8% were female, and 42.2% were male. The etiology was identified as gallstones in 64.1% of the cases, while in 31.2%, no specific cause was found and was considered idiopathic. Only one of the patients reported a history of alcohol consumption. Abdominal computed tomography (CT) was used in 93.6% of the patients, 42.2% had hypertension, and 15.6% had diabetes mellitus as an additional disease. There were no additional diseases in 34.4% of cases. The bedside index of severity in AP and sepsis and septic shock criteria scores were calculated for all the patients. The average length of hospital stay was 4 days. Table 1 presents the patient’s demographic and clinical characteristics.

**Table 1:** Demographic and clinical characteristics of participants.

Numeric variables	n	Min.	Max.	Mean	SD
Age	64	19	99	62.66	17.67
Body mass index	64	19.0	36.9	26.45	2.93
Hospitalization duration	64	1	18	4.36	2.68
BISAP score	64	0	2	0.78	0.63
SIRS score	64	0	4	0.58	0.75

BISAP = bedside index of severity in acute pancreatitis, SIRS = sepsis and septic shock criteria.

According to the Revised Atlanta Severity Scoring for the patients included in the study, 35 patients (54.7%) had mild pancreatitis, 12 (18.8%) had moderate pancreatitis, and 17 (26.6%) had severe pancreatitis. On the first day, the difference in SAL-β averages between individuals with mild and moderate pancreatitis and healthy individuals was not statistically significant (*P* > .05). However, the difference in SAL-β averages between individuals with severe pancreatitis and healthy individuals was significant (*P* < .05). The SAL-β averages of the individuals with severe pancreatitis were higher (Table 2).

**Table 2:** Comparison of 1st-day salusin beta averages in patients and healthy individuals according to Atlanta severity.

	Patient group			Control group			Significance
	n	Mean	SD	n	Mean	SD	
Mild	35	105.91	51.09	18	112.88	32.60	*U* = 303.000, *P* = .882
Moderate	12	126.96	29.10	18	112.88	32.60	*U* = 83.000, *P* = .290
Severe	17	340.41	271.52	18	112.88	32.60	*U* = 27.000, *P* = .000

The difference in the mean values of WBC, NLR, MPV, CRP, sedimentation, amylase, and lipase between the 1st and 3rd days was not statistically significant (*P* > .05) (Table 3). There was no significant relationship between SAL-β levels and the mean values of WBC, NLR, MPV, CRP, sedimentation, amylase, or lipase on the 1st and 3rd days (*P* > .05) (Table 4).

**Table 3:** Distribution of laboratory findings between the 1st and 3rd days.

Parameters	n	First day values	Third day values
WBC (10^3^/UL)	64	11924.69	11485.38
NLO	64	12.49	5.2586
MPV (fL)	64	10.05	10.17
CRP (mg/L)	64	49.41	85.46
Sedimentation	64	24.27	28.94
Amylase (U/L)	64	1420.08	230.19
Lipase (U/L)	64	2927.66	232.07

CRP = sensitive C-reactive protein, MPV = mean platelet volume, WBC = white blood cell count.

**Table 4:** Investigation of the relationship between salusin beta levels and laboratory findings on the 1st and 3rd days.

		Salusin beta (1st day)	Salusin beta (3rd day)
	*r*	−0.035	−0.126
WBC (10^3^/UL)	*P*	.785	.319
	n	64	64
	*r*	0.192	−0.077
NLO	*P*	.129	.547
	n	64	64
	*r*	−0.044	−0.034
MPV (fL)	*P*	.731	.792
	n	64	64
	*r*	−0.048	−0.136
CRP (mg/L)	*P*	.704	.284
	n	64	64
	*r*	−0.228	−0.097
Sedimentation	*P*	.070	.446
	n	64	64
	*r*	−0.009	−0.037
Amylase (U/L)	*P*	.946	.774
	n	64	64

CRP = sensitive C-reactive protein, MPV = mean platelet volume, *r* = Spearman correlation test, WBC = white blood cell count.

Both on the 1st and 3rd days, the difference in mean SAL-β levels was statistically significant (*P* < .05). In the analysis conducted to determine which group contributed to this difference (U), it was found that on the 1st day, individuals with severe pancreatitis, according to the Revised Atlanta Scoring, had higher SAL-β averages than individuals with mild and moderate pancreatitis. Similarly, on the 3rd day, individuals with severe pancreatitis had higher SAL-β averages than those with mild and moderate pancreatitis (Table 5).

**Table 5:** Comparison of salusin beta averages according to Atlanta severity.

		n	Mean	SD	Significance
1st day	Mild* Moderate† Severe‡	35 12 17	105.91 126.96 340.41	51.09 29.10 271.52	*X*^2^ KW = 23.938 *P* = .000 c > a,b
3rd day	Mild* Moderate† Severe‡	35 12 17	104.03 115.85 367.78	42.78 35.88 294.48	*X*^2^ KW = 26.305 *P* = .000 c > a,b

*X*^2^ KW = Kruskal–Wallis test.

*a; †b; ‡c.

There was no statistically significant relationship between the Atlanta severity level and WBC, NLR, MPV, CRP, and Amylase levels on day 1st (*P* > .05). Just like on the 1st day, there was no statistically significant relationship between the Atlanta severity level and WBC, MPV, CRP, and Amylase levels on the 3rd day (*P* > .05). On day 3rd, there was a statistically significant, positive, and low-level relationship between Atlanta severity levels and NLR (*P* < .05) (Table 6).

**Table 6:** Relationship between Atlanta severity levels and other markers on the 1st and 3rd days.

		1st days	3rd days
WBC	*r* *P* n	0.048 .706 64	0.047 .711 64
NLR	*r* *P* n	0.148 .244 64	0.263 .036 64
MPV	*r* *P* n	−0.138 .278 64	−0.093 .464 64
CRP	*r* *P* n	0.057 .657 64	0.099 .435 64
Amylase	*r* *P* n	0.001 .993 64	−0.010 .937 64

CRP = sensitive C-reactive protein, MPV = mean platelet volume, NLR = neutrophil-lymphocyte ratio, *r* = Spearman correlation test, WBC = white blood cell count.

**Figure 1.**
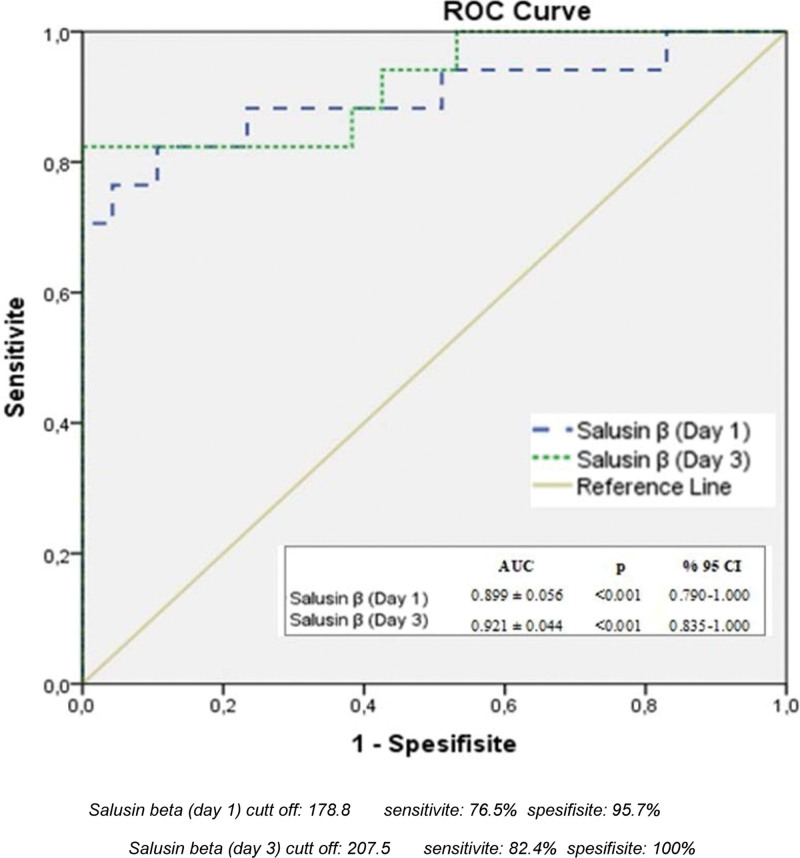
ROC analysis plot showing prediction of severe pancreatitis (Revised Atlanta Classification) according to salusin beta 1st and 3rd day values. ROC = receiver operating characteristic.

According to the receiver operating characteristic analysis using the Youden Index, the cutoff value for SAL-β on the 1st day was 178.8 pg/mL, and on the 3rd day, it was found to be 207.5 pg/mL (Fig. 1).

## 4. Discussion

AP is an acute inflammatory disease of the pancreas that can affect adjacent and distant organs to varying degrees. While most cases manifest as a mild form and resolve without leaving lasting effects, some can progress more severely, leading to the development of local and systemic complications and, in some instances, mortality. Severe pancreatitis remains a challenging disease for early diagnosis in clinical practice, with a high mortality rate. Reperfusion of damaged tissues and the increased release of inflammatory cytokines and free radicals contribute to systemic damage. Imaging methods, scoring systems, and biochemical parameters are commonly used to predict the AP severity of AP. However, there is a need for new biomarkers to complement existing scoring systems. Salusins are secreted in blood vessels, monocytes, and macrophages and can be found in human body fluids. Salusins stimulate the proliferation of VSMCs and fibroblasts. In particular, SAL-β, has various functions, including cytokine function, modulation of vascular inflammation, and oxidative damage. Our study is significant as it is the first to evaluate SAL-β levels in patients with AP.^[8–12]^

While most cases of AP follow a mild course, severe forms demand increased attention due to their high morbidity and mortality. Several single and multiparametric scoring systems have been defined to assess the severity of the disease by, incorporating laboratory parameters and physiological and radiological assessments. Utilizing these scoring systems for evaluation may not be clinically practical, emphasizing the need for an easy and accurate method for predicting severity. Approximately 23% of deaths related to AP occur within the first 3 days, and 53% occur within the first week.^[13]^ Hence, in our study, we investigated whether serum SAL-β levels could be useful in predicting the AP severity of AP.

Proinflammatory cytokines play a crucial role in the pathogenesis of AP and systemic complications. SAL-β, an endogenous neuropeptide, participates in various functions, including vascular inflammation, modulation of the cardiovascular system, cytokine function, and oxidative damage. In particular, SAL-β is a potential pro-oxidant in cardiomyocytes, VSMCs, endothelial cells, and renal cells, suggesting that it may be a critical regulator of oxidative stress-related diseases such as severe pancreatitis. Portosplenomesenteric venous thrombosis occurs in approximately half of the patients with acute necrotizing pancreatitis, and in severe cases, hypotension may develop secondary to low peripheral vascular resistance and hypovolemia. Assuming that SAL-β concentration is positively correlated with coronary artery injury or stenosis, a potential marker of atherosclerosis, and may increase the secretion of inflammatory factors, it may be elevated in severe pancreatitis with vascular complications.^[14–18]^

Elden et al^[19]^ found a significantly higher median value of SAL-β in patients with sudden hearing loss than in the control group. They suggested that SAL-β may be considered a poor prognostic factor. In a study by Hajialilo et al^[20]^ involving patients diagnosed with systemic lupus erythematosus (SLE), serum SAL-β levels were significantly higher than those in control groups. Patients with nephritis and thrombosis had significantly higher serum SAL-β levels. These findings indicate a potential role for SAL-β in the pathogenesis of SLE, particularly as a biomarker for nephritis and thrombosis in SLE. In a study by Cakir et al,^[21]^ which focused on inflammation, demyelination, reactive gliosis, and neuronal damage characterizing multiple sclerosis (MS) as a potentially progressive autoimmune disorder of the central nervous system, the relationship between salusin alpha and SAL-β peptides and inflammation, as well as their association with relapsing-remitting MS (RRMS), was investigated. Salusin alpha and beta are widely expressed in various tissues, including the central nervous system. The study revealed significantly elevated levels of salusin alpha and SAL-β in RRMS patients compared to those in healthy controls. A strong positive correlation was found between salusin alpha and SAL-β levels, indicating an association between salusin alpha, SAL-β, and MS. As RRMS represents the initial stage and the most common type of MS, conducting biomarker studies during this period is crucial for early treatment planning. In summary, these 3 studies suggested that these biomarkers can be used to detect the severity of inflammation and thrombosis in pancreatitis.

In diabetic cardiomyopathy, it has been demonstrated that SAL-β leads to inflammation, and its inhibition can potentially reduce heart dysfunction, oxidative stress, and inflammation in diabetic cardiomyopathy. Li et al proposed the potential beneficial effects of SAL-β -blockade in essential hypertension, suggesting the downregulation of inflammatory molecules and reduction of oxidative stress through this mechanism. Based on these studies, the use of SAL-β as an independent prognostic biomarker for severe pancreatitis can be considered. Additionally, considering studies reporting that SAL-β inhibition alleviates oxidative stress and inflammation in diabetic rats, inhibition of SAL-β could be explored as an additional treatment for severe pancreatitis.^[11,22]^

The use of serum endocan levels as prognostic indicators in patients with pancreatitis has been demonstrated. In a study by Albayrak et al, endocan levels, secreted by endothelial cells in response to inflammatory cytokines, were shown to be useful in determining the prevalence of ulcerative colitis characterized by mucosal inflammation and could be beneficial in treatment planning.^[23,24]^ Elevated levels of endocan, similar to SAL-β, were found in both patients with AP and severe ulcerative colitis, aligned with the design of our study. Ultimately, biomarkers identified as elevated in severe pancreatitis can be used for prognosis monitoring and assist clinicians.

In AP, radiological scoring systems, clinical scores, neutrophil-to-lymphocyte ratio, and serum C-reactive protein level can be used to assess severity and mortality.^[25]^ Approximately 20% of patients with AP develop severe AP, which may be associated with the dysfunction of multiple organs (respiratory, cardiovascular, and renal).^[26]^ Imaging methods are crucial for the diagnosis, prognosis, and demonstration of complications in AP. CT is the most important imaging method for diagnosis and demonstrating intra-abdominal complications. CT findings of AP cover a wide range from mild pancreatitis to necrosis. The sensitivity of CT in the diagnosis of AP is approximately 90%, while its specificity has increased to 92%. Dynamic contrast-enhanced CT is the most effective method for diagnosing AP and for demonstrating necrosis. The sensitivity of this test for the prognosis of AP exceeded 90%. In our study, contrast-enhanced abdominal CT was performed in most patients (90%) with appropriate renal function conditions. Therefore, contrast-enhanced radiological assessment may not always be possible owing to nephrotoxicity. Other scoring systems may also contribute to the evaluation of severity and mortality. Therefore, contrast-enhanced radiological assessment may not always be possible owing to nephrotoxicity. Other scoring systems may also contribute to the evaluation of severity and mortality. In our study, we used these scoring systems; however, there was no statistical difference between the stages of pancreatitis. CRP levels can be influenced by various factors.^[27–29]^ Therefore, there is a need for more specific markers such as SAL-β.

Despite recent advances in understanding the pathophysiology of AP, further research is needed to enable faster and more accurate prediction of severe AP. Moreover, several clinical and critical care scores can help retrospectively determine the severity and mortality of AP (e.g., Ranson score, Balthazar score, SOFA score, APACHE II score, and Marshall score). However, none of these scores are widely applied in clinical routines outside of clinical research. These scoring systems are not sufficient to predict the severity of pancreatitis.^[30–32]^

It has been shown in many studies that MPV and NLR increase inflammatory conditions. These commonly used parameters are also markers reflecting inflammation that can be used as a risk indicator in atherosclerosis-related diseases. For example, a significant relationship was found between NLR, average platelet volume, and fibrosis in chronic hepatitis B patients. In another study, MPV values were found to be higher in lung cancer patients with metastasis than in lung cancer patients without metastasis. In addition, NLR can be used to predict the severity of AP, considering its limited accuracy in acute and/or resource-limited settings. Therefore, since these parameters used in daily practice are increased in many diseases, their use may not be safe in predicting severe pancreatitis, just like CRP, as seen in our results.^[12,33–35]^

The main limitation of our study was its small sample size. No statistically significant differences were found between the mild and moderate pancreatitis groups. We believe that the small sample size of our study may have contributed to this statistical insignificance. However, we believe that SAL-β may also contribute to the monitoring of severe pancreatitis.

In conclusion, considering the risk of mortality and morbidity in AP, the ability to predict patient prognosis in advance is crucial. The serum SAL-β level in patients with AP is the subject of the first study in the literature, and there is a significant relationship between SAL-β levels and the severity of AP. SAL-β can be used to detect and monitor the presence of severe pancreatitis. Further extensive clinical studies with larger case series are needed.

## Author contributions

**Conceptualization:** Bulent Albayrak.

**Data curation:** Bulent Albayrak, Emel Ayvaz Guneyin, Muhammet Celik.

**Formal analysis:** Bulent Albayrak, Emel Ayvaz Guneyin, Muhammet Celik.

**Funding acquisition:** Bulent Albayrak, Emel Ayvaz Guneyin, Muhammet Celik.

**Investigation:** Bulent Albayrak, Emel Ayvaz Guneyin.

**Methodology:** Bulent Albayrak, Emel Ayvaz Guneyin, Muhammet Celik.

**Project administration:** Bulent Albayrak.

**Resources:** Bulent Albayrak, Emel Ayvaz Guneyin, Muhammet Celik.

**Software:** Bulent Albayrak, Muhammet Celik.

**Supervision:** Bulent Albayrak, Emel Ayvaz Guneyin.

**Validation:** Bulent Albayrak, Emel Ayvaz Guneyin, Muhammet Celik.

**Visualization:** Bulent Albayrak, Muhammet Celik.

**Writing – original draft:** Bulent Albayrak, Emel Ayvaz Guneyin, Muhammet Celik.

**Writing – review & editing:** Bulent Albayrak.
